# Multimodal Deep Learning Differentiates Papilledema and Non-Arteritic Anterior Ischemic Optic Neuropathy From Healthy Eyes

**DOI:** 10.1167/iovs.67.1.12

**Published:** 2026-01-07

**Authors:** David Szanto, Asala Erekat, Brian Woods, Jui-Kai Wang, Mona Garvin, Brett Johnson, Randy Kardon, Michael Wall, Edward Linton, Mark J. Kupersmith

**Affiliations:** 1 Department of Ophthalmology, New York Eye and Ear Infirmary at Mount Sinai, New York, New York, United States; 2 Department of Neurology, Icahn School of Medicine at Mount Sinai, New York, New York, United States; 3 Irish Clinical Academic Training Programme, Department of Ophthalmology, Galway University Hospital, Galway, Ireland; 4 Physics Department, School of Natural Sciences, University of Galway, Galway, Ireland; 5 Department of Ophthalmology, University of Texas Southwestern Medical Center, Dallas, Texas, United States; 6 Center for the Prevention and Treatment of Visual Loss, Iowa City VA Health System, Iowa City, Iowa, United States; 7 Department of Electrical and Computer Engineering, University of Iowa, Iowa City, Iowa, United States; 8 Department of Ophthalmology and Visual Sciences, University of Iowa, Iowa City, Iowa, United States; 9 University of Iowa Carver College of Medicine, Iowa City, Iowa, United States; 10 University of Iowa Hospitals and Clinics, Iowa City, Iowa, United States

**Keywords:** corneal stroma, fibrosis, TSP1, A2M, CGRP

## Abstract

**Purpose:**

Optic nerve head (ONH) swelling, a critical feature in idiopathic intracranial hypertension (IIH) and non-arteritic anterior ischemic optic neuropathy (NAION), can present diagnostic challenges. We explored a multimodal deep learning (DL) approach integrating optical coherence tomography (OCT) scans and fundus photographs to enhance diagnostic accuracy for differentiating IIH, NAION, and healthy eyes.

**Methods:**

We developed two separate models using 7019 OCT scans (3D-ResNet-18) and 17,657 fundus photos (ResNet-50) to classify eyes with papilledema (2315 OCT, 6349 fundus), NAION (841 OCT, 1814 fundus), and healthy eyes (3863 OCT, 9494 fundus). We arranged the dataset so that the test set consisted entirely of same-day OCT scans and fundus photos, with each modality (OCT and fundus) contributing at least 15% of the data for each class. We combined output probabilities from both models using two methods: an F1-weighted sum by class (F1WS), as well as an XGBoost model. Performance of each was evaluated with AUC-ROC, accuracy, precision, recall, and F1 scores.

**Results:**

The OCT model alone achieved a test accuracy of 93.5%, with the fundus photo model reaching 93.9%. The multimodal F1WS and XGBoost models achieved an accuracy of 97.5% and 98.3%, respectively.

**Conclusions:**

Combining OCT and fundus photographs improves the classification of IIH, NAION, and healthy eyes, showing the value of using complementary imaging modalities. This approach supports the use of DL to aid diagnosis and clinical management of optic nerve head swelling. It may also be extended to leverage DL from additional data sources, such as macular scans or visual field tests.

Optic nerve head (ONH) swelling occurs in a number of optic nerve and ophthalmic disorders as a result of different mechanisms. The etiologies include idiopathic intracranial hypertension (IIH) and non-arteritic anterior ischemic optic neuropathy (NAION). Both IIH and NAION affect the ONH but arise from distinct pathophysiological processes and require different clinical management strategies and testing.

NAION is the most common acute optic neuropathy in individuals over the age of 50.^1^^,^^2^ NAION almost always presents unilaterally with acute, painless vision loss and characteristic optic disc swelling, cotton wool spots, and peripapillary hemorrhages.^3^^–^^5^ Visual outcome in NAION is typically poor, with limited recovery of the visual field in most cases and no proven therapy. Accurate diagnosis is crucial for prognosis and patient counseling, and avoiding emergency room referral, extensive neuro-imaging, and unproven therapies.

In contrast, IIH primarily affects younger overweight women, and is characterized by elevated intracranial pressure that causes papilledema and vision perturbation.^6^ Diagnosing IIH can be challenging when visual loss is acutely reported. Papilledema can also be due to other causes of intracranial hypertension, some of which can be life-threatening. Once IIH is suspected, neuro-imaging, including a contrast magnetic resonance imaging and venogram, as well as a lumbar puncture are typically performed to confirm elevated intracranial pressure and rule out other conditions. Weight management and acetazolamide in escalating doses, CSF shunting, optic nerve sheath fenestration, or dural venous sinus stenting are used depending on the degree of vision loss or risk of visual loss.

Although IIH and NAION are distinct entities, they can share similar pathologic ONH features, including swelling, cotton wool spots, hemorrhages, venous dilation, and peripapillary retinal folds and distortions, making accurate differentiation a challenge by ophthalmoscopy alone.^7^^,^^8^ This may be an issue in unilateral cases when papilledema in IIH is highly asymmetric; this occurs in 10% of cases.^6^ In cases where both eyes are affected or in sequential NAION, the clinical picture can become even more challenging, requiring reliance on a combination of clinical assessments, imaging features, and clinician expertise to reach a diagnosis. This differentiation is crucial, as management strategies for IIH and NAION differ substantially, with IIH requiring intracranial pressure management and NAION focusing on addressing systemic risk factors and managing visual symptoms.

The emergence of artificial intelligence (AI) and deep learning (DL) has created unprecedented opportunities to aid diagnostic accuracy in ophthalmology, particularly through automated image analysis. DL models applied to retinal images have achieved remarkable results in identifying and classifying common ophthalmic conditions from diabetic retinopathy to glaucoma to age-related macular degeneration and beyond.^9^^–^^21^ By leveraging these AI tools, it is now possible to detect intricate image patterns beyond human perception, suggesting that DL could also support the challenging task of differentiating neuro-ophthalmic conditions that swell the ONH like IIH and NAION. Most AI models in ophthalmology, however, rely on a single imaging modality, typically fundus photography or optical coherence tomography B-scans (OCT).^22^ Our previous work demonstrated significant potential in leveraging the full unsegmented three-dimensional (3D) OCT volume to differentiate between various causes of ONH swelling^23^ or atrophy,^24^ as well as the ability of using traditional fundus photos to differentiate the same swelling conditions.^25^

In eyes where differentiating subtle ONH changes is particularly challenging, a multimodal approach may hold greater promise by incorporating multiple perspectives on ONH morphology. Fundus photography and OCT provide complementary information that may be especially valuable in differentiating causes of ONH swelling. Fundus photos offer a broad view of the optic disc and surrounding retina, whereas OCT provides high-resolution, cross-sectional images of the deeper retinal layers and structure, which provide a three dimensional view of the ONH. By combining fundus and OCT data, a multimodal DL model has the potential to capture nuanced differences in ONH appearance between papilledema, NAION, and healthy controls, which might be missed by single-modality models. Currently, no model integrates the full OCT volume with fundus photos, and multimodal approaches involving OCT and fundus photos are not yet used for differentiating causes of ONH swelling.

We propose a novel multimodal DL model that integrates both fundus photographs and unsegmented 3D ONH OCTs to distinguish between papilledema caused by intracranial hypertension, NAION, and healthy ONH presentations. We hypothesize that this multimodal model will outperform single-modality models, providing a robust tool for automated diagnosis of ONH swelling causes and potentially offering new insights into the complex pathologies underlying IIH and NAION.

## Methods

This study was approved by the Institutional Review Board of the Icahn School of Medicine at Mount Sinai and did not require additional consent because all data were de-identified and collected from participants who had consented to data use across multiple study institutions. The study adhered to the tenets of the Declaration of Helsinki and HIPAA regulations. During the preparation of this work the authors used ChatGPT-4o to assist with refining the language and structure of the content. After using this tool/service, the authors reviewed and edited the content as needed and take full responsibility for the content of the publication. The datasets generated or analyzed during the current study are not publicly available because of the Health Insurance Portability and Accountability Act (HIPAA) but are available from the corresponding author on reasonable request. The underlying code for this study is not publicly available but may be made available to qualified researchers on reasonable request from the corresponding author. All data sources and demographic characteristics by disease are summarized in Table 1.

**Table 1.** Demographic Information for Eyes With Idiopathic Intracranial Hypertension, Non-Arteritic Anterior Ischemic Optic Neuropathy, and Healthy Eyes, Split By Modality

	Eyes	Scans	Age	Male
OCT				
IIH (IIHTT)	220	537	29.0 ± 7.4	4%
IIH (Iowa Clinic)	70	313	26.7 ± 11.8	20%
IIH (NYEE)	184	342	27.6 ± 8.9	16%
IIH (Sinai Clinic)	296	1123	33.5 ± 12.1	18%
NAION (Iowa Clinic)	109	114	58.0 ± 13.0	68%
NAION (NYEE)	39	54	61.6 ± 12.4	62%
NAION (QRK207)	344	596	61.0 ± 7.8	75%
NAION (Sinai Clinic)	38	77	63.8 ± 12.6	64%
Healthy (Athlete)	254	913	20.1 ± 1.4	100%
Healthy (Iowa Clinic)	540	567	46.1 ± 16.7	32%
Healthy (QRK207)	352	1178	61.2 ± 7.4	70%
Healthy (Sinai Clinic)	408	463	43.9 ± 17.9	22%
Healthy (VIPII)	109	742	61.2 ± 8.9	37%
Photo				
IIH (IIHTT)	310	5803	28.9 ± 7.6	3%
IIH (Iowa Clinic)	108	158	31.9 ± 11.0	13%
IIH (NYEE)	383	388	32.7 ± 12.0	10%
NAION (Iowa Clinic)	80	80	57.6 ± 13.3	69%
NAION (NYEE)	67	265	61.3 ± 10.2	62%
NAION (QRK207)	699	1469	61.3 ± 7.7	69%
Healthy (DRD)	4129	4129		
Healthy (Iowa Clinic)	540	1447	46.1 ± 16.7	32%
Healthy (ORIGA)	473	473		
Healthy (QRK207)	539	3445	60.9 ± 7.4	67%

Healthy eyes from the DRD grade 0 and the ORIGA datasets lacked demographic information.

Demographics of participants.

### OCT

Our OCT datasets consist of Cirrus SD-OCT 200 × 1024 × 200 (6 × 2 × 6 mm^3^) ONH volume scans (Zeiss-Meditec, Inc, Dublin, CA, USA) from three categories: IIH, NAION, and healthy controls.

#### IIH

We sourced IIH OCT scans from four groups of patients. The participants in the IIH Treatment Trial (IIHTT) had mild visual field loss and were randomized to either acetazolamide and diet or placebo and diet.^26^ We excluded eyes with grade 0 or resolved papilledema, utilizing scans with a corresponding photo with Frisén grade ≥ 1 across different time points, yielding a total of 537 scans. Additional data were obtained from the Mount Sinai Neuro-Ophthalmology Service, contributing 1123 scans, including 114 cases of papilledema secondary to other causes such as intracranial tumors or dural venous sinus thrombosis. These were retained given the shared pathophysiology with IIH-related papilledema. We also included IIH patient OCTs with 342 scans from New York Eye and Ear Infirmary (NYEE) and an additional 313 scans from the University of Iowa neuro-ophthalmology clinic, featuring Frisén grades 4–5 papilledema because of IIH to have enough severe cases for study, all of which had an RNFL ≥ 110 µm (which exceeds the normal average RNFL thickness).

#### NAION

We obtained OCT scans from the QRK207 trial, a multi-national trial involving participants aged 50-80 years diagnosed with NAION within 14 days of vision loss.^27^^–^^29^ The dataset included 596 ONH OCT scans of acute NAION, supplemented with 52 additional scans from the Mount Sinai neuro-ophthalmology clinic. We also used OCTs of eyes with acute NAION, including 54 scans from NYEE and 111 scans from the University of Iowa neuro-ophthalmology clinic, all of which using RNFL ≥ 110 µm to exclude eyes with an atrophic ONH.

#### Healthy Eyes

We included OCT data from a variety of demographics. The first was from a study which examined variability across perimetric stimuli of different sizes and the ability to distinguish between healthy and impaired visual fields in glaucoma patients. The control group included 60 healthy individuals with no eye disease, who underwent OCT scans every six months, totaling 742 scans.^30^ The second source included scans of the University of Iowa athletes which had 913 scans from participants aged comparably to the IIH group. We also incorporated 1178 scans from normal fellow eyes of participants in the QRK207 trial, excluding eyes with prior NAION.^27^^–^^29^ Additional sources comprised 463 scans from the Mount Sinai Neuro-Ophthalmology clinic involving patients diagnosed with headaches but without optic nerve abnormalities, as well as 567 scans from visits of patients without optic disc edema at the University of Iowa Neuro-Ophthalmology clinic.

### Fundus Photos

#### IIH

We collected IIH fundus photos from the IIHTT, which included disc edema grades 1–5, with a total of 5803 photos collected at multiple study visits.^26^ Additionally, we obtained 158 images from the University of Iowa's Neuro-Ophthalmology clinic (Topcon RC 50-DX retinal camera with a Megavision 6 megapixel back; Topcon Optical Company, Tokyo, Japan) and 388 similar images from NYEE (Topcon TRC 50IX; Topcon Optical Company) with Frisén grade ≥ 1 or same-day OCT with RNFL ≥ 110 µm.

#### NAION

We used the QRK207 trial fundus photos of study eyes at day 1 of enrollment resulting in 1472 high-resolution images taken with a standard single-lens reflex system.^27^^–^^29^ In addition, we included 265 photos from NYEE and 80 from the University of Iowa Neuro-Ophthalmology Clinic, all of which had corresponding OCTs with an RNFL thickness of ≥110 µm.

#### Healthy Eyes

We sourced healthy images from the EyePACS diabetic retinopathy dataset (DRD), using those with no diabetic retinopathy, termed grade 0.^31^ These photos were captured across multiple sites, including the EyePACS tele-ophthalmology platform in the United States and three eye hospitals in India. Demographics for these participants are not public. We also collected 473 photos from the ORIGA dataset, which contained high resolution, ONH-centered images of healthy eyes aged 40-80.^32^

We used 3445 photos of healthy eyes from the fellow eyes in the QRK207 trial, excluding those with a history of NAION.^27^^–^^29^ Additionally, we incorporated 1447 fundus photographs from healthy participants without optic nerve or retinal pathology who underwent eye examinations at the University of Iowa Neuro-Ophthalmology Clinic.

### Pre-Processing

To reduce computational load, OCT volumes were downscaled to 100 × 256 × 100. No other OCT preprocessing was performed.

**Figure 1.**
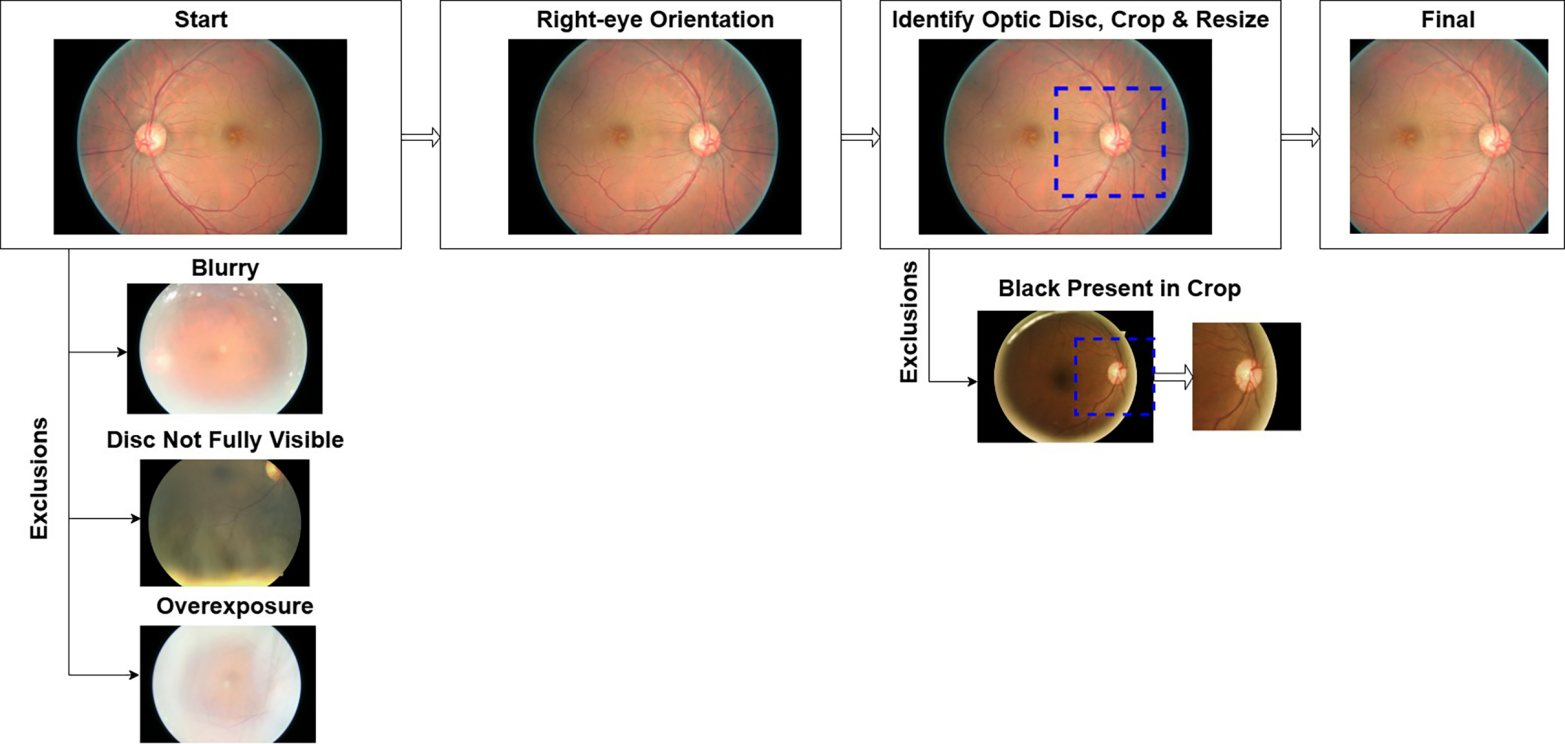
**Fundus photo preprocessing pipeline.** We standardized fundus photos by horizontally flipping left-eye images to right-eye orientation, locating and cropping around the optic disc, and resizing. We excluded low-quality photos and photos where the optic disc was not centered, ensuring consistency for our deep learning architecture. Reprinted from *American Journal of Ophthalmology*, Vol. 276, Szanto D, Erekat A, Woods B, Wang JK, Garvin M, Johnson BA, Kardon R, Linton E, Kupersmith MJ. Deep Learning Approach Readily Differentiates Papilledema, Non-Arteritic Anterior Ischemic Optic Neuropathy, and Healthy Eyes, pp. 99–108, Copyright © 2025, with permission from Elsevier.

Because of variations in fundus photo resolution and field of view, all images were standardized to an optic-disc centered view as described in our prior work (Fig. 1).^25^ We horizontally flipped left-eye images to right-eye orientation. We cropped photos to square dimensions centered on the optic disc, with each side at 60% of the shortest image dimension. We excluded images that were blurry, over- or underexposed, or whose optic nerves extended outside the field of view. Remaining images were resized to 256 × 256 pixels, preserving the central optic disc region while incorporating peripheral retina context. The 60% determination was a compromise between making sure there was enough retinal detail for classification while also minimizing artifacts that could bias the model.

### Data Organization

**Figure 2.**
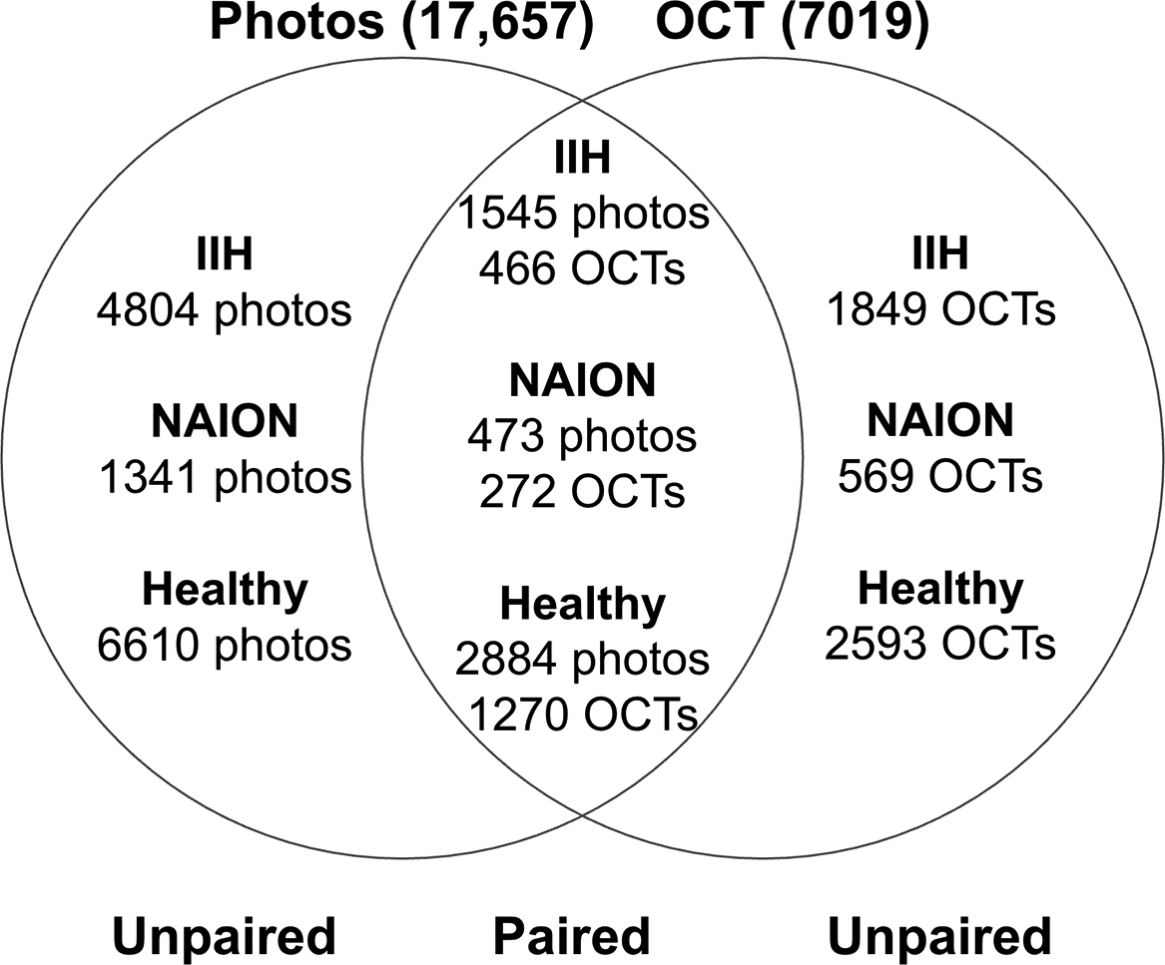
**Overlap of same-day and non-same day fundus photos and OCTs.** Description of overlap between paired (same-day) and unpaired (non-same-day) fundus photos and OCTs with a patient-level split. The overlapping middle section indicates the number of paired data. The nonoverlapping areas represent data where photos or OCTs were collected on different days. Multiple scans were obtained for each modality on the same day, resulting in varying numbers across paired and unpaired data points.

Figure 2 details count of same-day (paired) and non-same-day (unpaired) OCTs and fundus photos. Paired data were obtained from IIHTT, Iowa Neuro-Ophthalmology Clinic, NYEE, and QRK207, whereas the remaining sites contributed only one imaging modality. We created two coordinated train/validation/test splits, one for fundus photographs and one for OCTs.

We first structured the test sets to contain paired OCT and fundus photos, allowing for a consistent evaluation of participants with first each modality independently and then in combination. However, to avoid data leakage (i.e., when scans from the same eye appear across the training, validation, or test sets), we also included other time points for the same eye that had unpaired data. We wanted to ensure that at least 15% of each class for each modality was represented in the test set. This approach, however, introduced challenges. There were substantial differences in the number of images or scans per modality for some eyes, depending on how the data were collected across different sources. As a result, enforcing a 15% representation for a class in one modality often led to much higher proportions for that class in the other modality. In IIH, participants often had fundus photographs collected at multiple time points, some when OCTs were not acquired (particularly in the IIHTT as OCTs were obtained at baseline and months 3 and 6, if collected at all). As a result, selecting 15% of the total IIH OCT data for the test set required including a larger portion of the corresponding fundus photographs, leading to 42% of the total IIH photo data being allocated to the test set. In contrast, in the QRK207 dataset for NAION, participants typically underwent OCT imaging at two acute time points, while only a single fundus photograph was usually captured early in the disease course. Consequently, the test set included 15% of the total NAION photo data, but this subset accounted for 32% of the total NAION OCT data. For the remaining data, which included all unpaired cases and paired data not allocated to the test set, we applied a 15% validation split grouped by eye to prevent data leakage. The rest of the data was used for training.

### Model Architecture

Our OCT model is derived from a fine-tuned 3D-ResNet-18^33^ architecture with a final output layer producing logits that are then transformed into class probabilities via a softmax function. We applied data augmentation during training, including normalization, random cropping with up to 10% removal from each dimension, repadding with black pixels, and Gaussian smoothing (σ = 0.2).

We created our fundus photo model from a fine-tuned ResNet-50^34^ architecture which also produces logits for each class that are softmaxed into class probabilities. Data augmentation included normalization using ImageNet means and standard deviations, random rotations, random cropping to a final size of 224 × 224 pixels, and color jittering.

For both models, we addressed class imbalance by weighting the loss functions inversely to the number of items in each class. This strategy helped prevent underrepresentation of less-frequent classes, such as NAION, and overrepresentation of more common classes, such as healthy eyes.

#### Multimodal Fusion Methods

To leverage the information provided by the OCT and fundus photo models, we explored two fusion methods that combined the output probabilities from each model. Our first method leveraged F1 weighted sums (F1WS) of the single-modality models. The output logits of each model were weighted based on the F1 score of each class obtained during testing. By emphasizing the contributions of each model according to its class-specific performance, this method aimed to balance the strengths of the two modalities. Photo and OCT weights for class *c* were calculated by the following:

$$\begin{array}{c} weights_{photo}\left[c\right]=\frac{F1_{photo}\left[c\right]}{F1_{photo}\left[c\right]+F1_{OCT}\left[c\right]} \end{array}$$

$$\begin{array}{c} weights_{OCT}\left[c\right]=\frac{F1_{OCT}\left[c\right]}{F1_{photo}\left[c\right]+F1_{OCT}\left[c\right]} \end{array}$$

And the final weighted sum of logits was calculated as:

$$\begin{array}{c} logits_{combined}\left[c\right]=weights_{photo}\left[c\right]*logits_{photo}\left[c\right]\\ +\;weights_{OCT}\left[c\right]*\;logits_{OCT}\left[c\right] \end{array}$$

The resulting logits were converted to probabilities using a softmax function to yield the final class prediction. This ensures that the modality better suited for detecting a specific disease is weighted more heavily in the final determination.

The second fusion method used a cross-validated XGBoost classifier trained on the output probabilities from both models, resulting in six input features (three class probabilities from each modality).^35^ A fivefold cross-validation was conducted within the test set, where the XGBoost model was trained on four folds and evaluated on the fifth. This process was repeated for all folds, ensuring the entire dataset was evaluated. This approach enabled the model to learn nonlinear relationships between the OCT and fundus photo predictions. To optimize the XGBoost model, we conducted a grid search during cross-validation, exploring a range of hyperparameters. We also analyzed feature importance by weight to assess the contribution of each input probability, enabling us to evaluate the relative influence of each modality on the final prediction.

The performance of the individual OCT and fundus photo models was first evaluated separately with AUC-ROC scores, overall accuracy, class-wide precision, recall, and F1 scores. We then applied the two fusion methods to assess the benefit of combining multimodal information, allowing for a comparative analysis of the independent and combined models, while also recalculating each performance metric. This setup enabled us to analyze both the overall performance and the modality-specific contributions to accuracy, thereby showcasing the value of a multimodal approach in differentiating between IIH, NAION, and healthy eyes. We used McNemar's test to compare the efficacy of the F1WS model with the XGBoost model to determine if one fusion method outperformed the other, as well as if they outperformed the unimodal models.

## Results

We investigated 7019 Cirrus OCT ONH volume scans from 2963 eyes and 17657 fundus photos from 7328 eyes. Tables 1 and 2 illustrate participant sources and demographics for each disease. We arranged our data as shown in Table 3. Model performances are found in Table 4.

**Table 2.** Racial Composition of Patients by Disease Category

Race	IIH	NAION	Healthy
American Indian or Alaska Native	1%	0%	0%
Asian	2%	18%	3%
Black or African American	28%	1%	7%
Multiracial	1%	0%	1%
Native Hawaiian or other Pacific Islander	0%	0%	0%
Other race	23%	7%	6%
Race unknown	4%	2%	6%
White	42%	72%	77%

Race information was not available for the DRD, ORIGA, or VIPII datasets. Because of rounding, percentages may not total exactly 100%.

Demographics of participants.

**Table 3.** The Test Set Consists Entirely of Paired Same-Day Fundus Photos and OCTs in a Patient-Level Split, With Unpaired Scans From Other Time Points Also Included to Prevent Data Leakage

	Train (OCT)	Val (OCT)	Test (OCT)	Train (Photo)	Val (Photo)	Test (Photo)
IIH	1601 (69.2%)	356 (15.4%)	358 (15.5%)	2726 (42.9%)	943 (14.9%)	2680 (42.2%)
NAION	446 (53.0%)	127 (15.1%)	268 (31.9%)	1278 (70.5%)	265 (14.6%)	271 (14.9%)
Healthy	2523 (65.3%)	572 (14.8%)	768 (19.9%)	6650 (70.0%)	1381 (14.5%)	1463 (15.4%)

Approximately 15% of the data from one modality with less data for that disease was allocated to the test set, whereas the other modality contained a larger proportion due to multiple scans or unpaired scans from other time points. Approximately 15% of the data was used for validation, whereas the remainder was allocated to training.

Data split by modality and diagnosis.

**Table 4.** Performance of the Four Models Created, Comparing Overall Accuracy, Average AUC for the ROC, and Macro-Average F1-Score for Each Class

	OCT	Photo	Multimodal (F1WS)	Multimodal (XGBoost)
Accuracy	93.5%	93.9%	97.5%	98.3%
Macro-AUCROC	0.980	0.988	0.998	0.997
F1: IIH	0.91	0.96	0.98	0.98
F1: NAION	0.90	0.81	0.95	0.97
F1: Healthy	0.96	0.93	0.98	0.99

The combined models outperformed the single-modality models in all metrics.

Performance metrics for all models.

**Figure 3.**
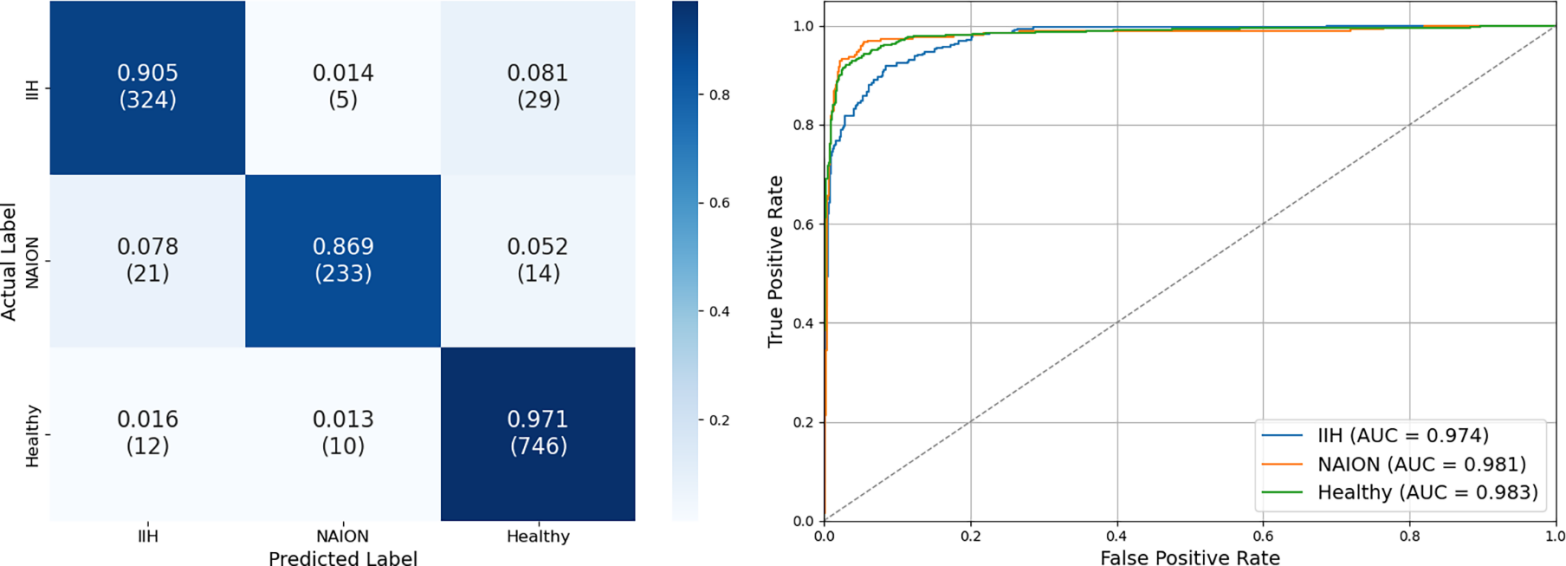
**OCT test set confusion matrix and ROC curve.** (*left*) Confusion matrix for test set optical coherence tomography optic nerve head scans by group. It demonstrates an overall accuracy of 93.5%, precision of 0.91, 0.94, and 0.95; recall of 0.91, 0.87, and 0.97; and F1 score of 0.91, 0.90, and 0.96 for eyes with idiopathic intracranial hypertension, non-arteritic anterior ischemic optic neuropathy, and healthy eyes, respectively. (*right*) The ROC curve showed high discriminatory power. The macro-average AUC score is 0.979.

Our OCT model achieved an overall test set accuracy of 93.5%. Test set AUC-ROC scores were 0.974, 0.981, and 0.983, precision was 0.91, 0.94, and 0.95, recall was 0.91, 0.87, and 0.97, and F1 scores were 0.91, 0.90, and 0.96 for IIH, NAION, and healthy eyes respectively (Fig. 3).

**Figure 4.**
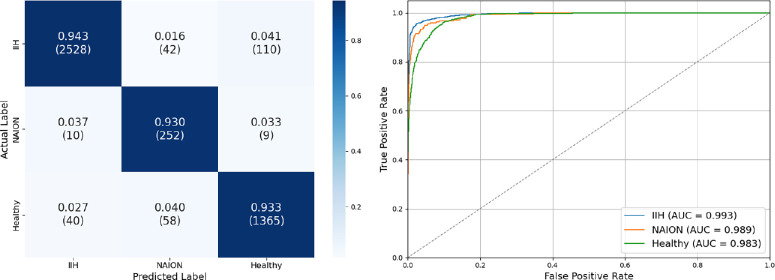
**Fundus test set confusion matrix and ROC curve.** (*left*) Confusion matrix for centered fundus photos by group. It demonstrates an overall accuracy of 93.9%, precision of 0.98, 0.72, and 0.92; recall of 0.94, 0.93, and 0.93; and F1 score of 0.96, 0.81, and 0.93 for eyes with idiopathic intracranial hypertension, non-arteritic anterior ischemic optic neuropathy, and healthy eyes, respectively. (*right*) The ROC curve showed high discriminatory power. The macro-average AUC score is 0.988.

Similarly, our fundus photo model achieved an overall test set accuracy of 93.9%. Test set AUC-ROC scores were 0.993, 0.989, and 0.983, precision was 0.98, 0.72, and 0.92, recall was 0.94, 0.93, and 0.93, and F1 scores were 0.96, 0.81, and 0.93 for IIH, NAION, and healthy eyes respectively (Fig. 4).

**Figure 5.**
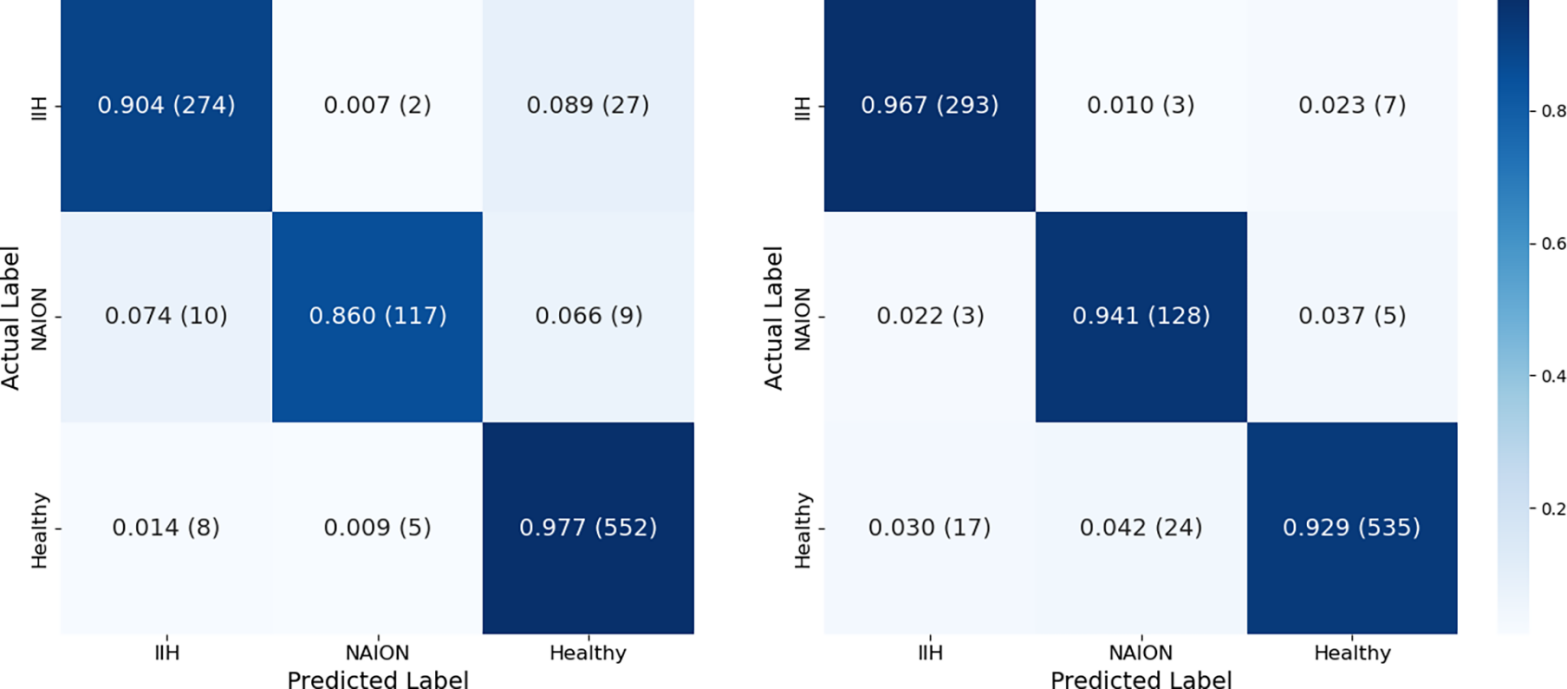
**Fundus photo and OCT classification test set for exclusively paired timepoints.** Performance comparison of two independent deep learning models, (*left*) one using OCT and (*right*) the other using fundus photos, for classifying idiopathic intracranial hypertension, non-arteritic anterior ischemic optic neuropathy, and healthy eyes. Here, we focus only on eyes with both an OCT and a fundus photo taken on the same day, allowing a direct comparison between the two models under identical clinical conditions. The pared down data showed similar results to the full test set, with an OCT accuracy of 93.9% and fundus photo accuracy of 94.2%.

After averaging logits for visits with multiple scans or photos and excluding unpaired measurements (because the fusion model inputs only accept one logit from each class), the OCT photo model had a test set accuracy of 93.9%, whereas the fundus photo model had an accuracy of 94.2%. AUC-ROC, precision, recall, and F1 scores remained very similar to that of the entire test set for both models (Fig. 5).

**Figure 6.**
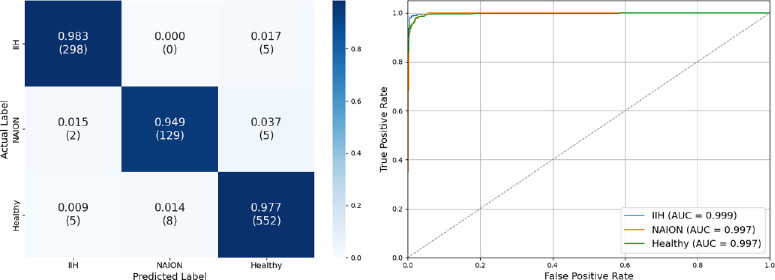
**Multimodal classification using F1-weighted logit summation.** Performance of our F1-weighted sum model that combines predictions for respective optical coherence tomography and fundus photo models. We assigned a weight to each model's output logits based on class-specific F1 score, ensuring that the modality better suited for detecting a specific disease contributes more to the final decision. (*left*) Our model demonstrates an overall accuracy of 97.5%, precision of 0.98, 0.94, and 0.98; recall of 0.98, 0.95, and 0.98; and F1 score of 0.98, 0.95, and 0.98 for eyes with idiopathic intracranial hypertension, non-arteritic anterior ischemic optic neuropathy, and healthy eyes, respectively. (*right*) The ROC curve had a macro-average AUC score of 0.998. All performance metrics exceed those of either modality alone.

Our F1WS multimodal model resulted in an accuracy of 97.5% with an AUC-ROC of 0.999, 0.997, and 0.997, as well as a precision of 0.98, 0.94, and 0.98, recall was 0.98, 0.95, and 0.98, and F1 scores were 0.98, 0.95, and 0.98 for IIH, NAION, and healthy eyes, respectively (Fig. 6).

**Figure 7.**
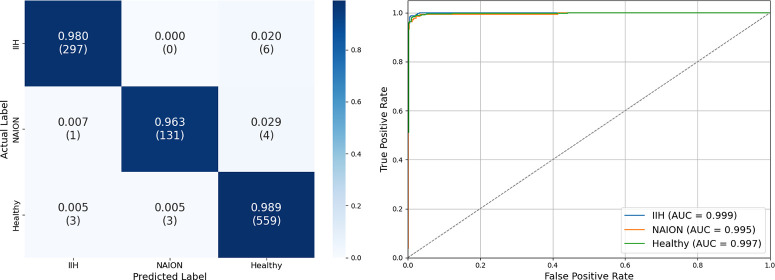
**Multimodal Classification Using Cross-Validated XGBoost.** Performance of an XGBoost model trained on the output class probabilities from independent optical coherence tomography and fundus photo models. We evaluated every item in the dataset using fivefold cross-validation, where the model was trained on four parts and tested on the fifth, repeating this process five times to ensure each sample contributed to both training and evaluation. (*left*) Our model demonstrates an overall accuracy of 98.3%, precision of 0.99, 0.98, and 0.98; recall of 0.98, 0.96, and 0.99; and F1 score of 0.98, 0.97, and 0.99 for eyes with idiopathic intracranial hypertension, non-arteritic anterior ischemic optic neuropathy, and healthy eyes, respectively. (*right*) The ROC curve had a macro-average AUC score of 0.997. All performance metrics exceed those of either modality alone.

**Figure 8.**
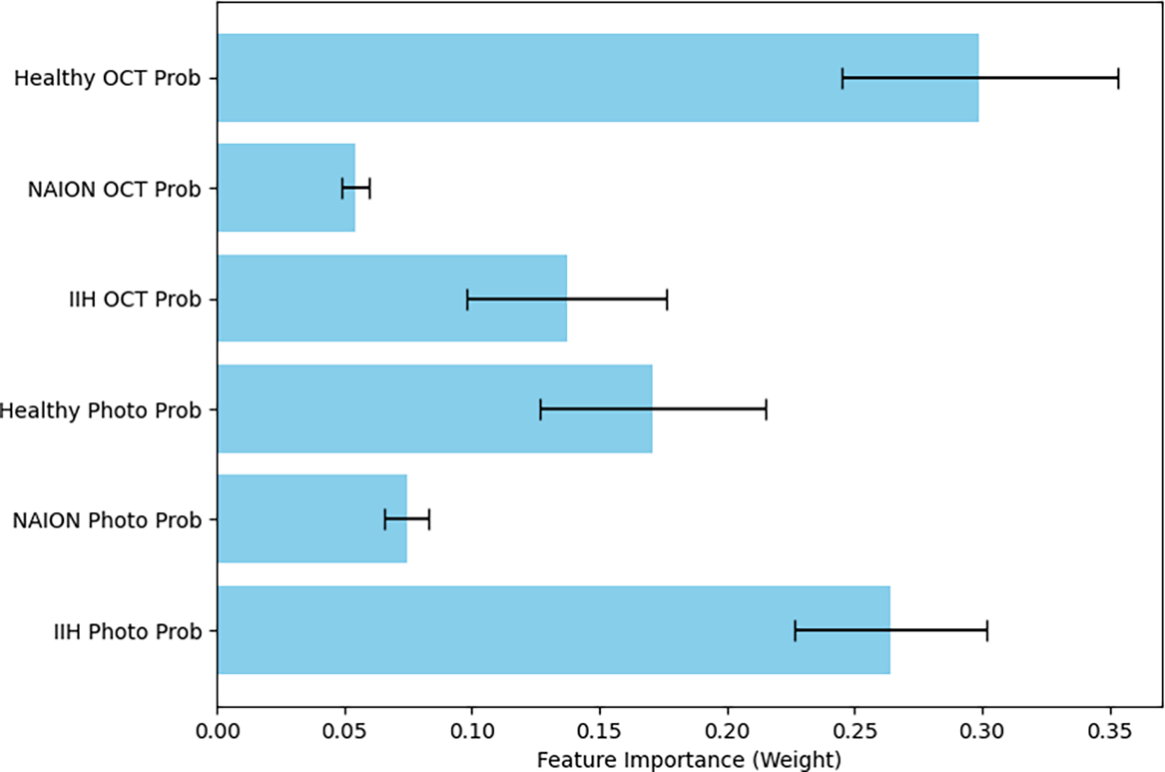
**Feature importance of OCT and fundus photo probabilities in XGBoost classification.** Feature importance of the XGBoost model based on output probabilities of the OCT and fundus photo models. *Error bars* indicate variability across cross-validation folds. OCT weights were more influential than fundus photo weights for healthy eyes, whereas fundus photo weights were more influential for both idiopathic intracranial hypertension and non-arteritic anterior ischemic optic neuropathy. In all cases, there was no overlap in the *error bars*.

The reported performance metrics are based on the XGBoost model trained using the optimal hyperparameters identified during cross-validation: a learning rate of 0.01, maximum depth of 3, 300 estimators, and full subsampling (colsample_bytree = 1.0, subsample = 1.0). The cross-validated hyperparameter-tuned XGBoost model resulted in an overall accuracy of 98.3% with an AUC-ROC of 0.999, 0.995, and 0.997, as well as a precision of 0.99, 0.98, and 0.98, recall was 0.98, 0.96, and 0.99, and F1 scores were 0.98, 0.97, and 0.99 for IIH, NAION, and healthy eyes respectively (Fig. 7).The feature importance analysis by weight revealed that OCT probabilities were more influential than fundus photo probabilities for healthy eyes, while fundus photo probabilities were more influential for both IIH and NAION (Fig. 8). In all cases, there was no overlap in the error bars.

There was insufficient evidence from McNemar's test to conclude that the performance of the F1WS and XGBoost models differed significantly (*P* = 0.12). However, there was a significant difference (*P* < 0.001) between each unimodal model and the multimodal models.

## Discussion

Our study demonstrates the significant diagnostic potential of a multimodal DL approach that integrates OCT volumes and fundus photographs for differentiating eyes with papilledema, NAION, and healthy eyes. By leveraging two types of imaging modalities, they had superior performance compared to individual OCT and fundus photo models, despite having a relatively smaller training size.

Our findings show that the multimodal fusion approaches (both F1WS and XGBoost-based fusion) outperformed single-modality models across all key performance metrics, including accuracy, AUC-ROC, and F1 scores. The F1WS method provides a computationally efficient and interpretable approach that performs similarly to the more complex XGBoost-based fusion model. Importantly, the superior performance of the multimodal models suggests that both OCT and fundus photographs contribute unique and complementary diagnostic information, enabling better differentiation of papilledema, NAION, and healthy eyes. No significant difference was observed between the multimodal model accuracy (*P* = 0.12), although a larger dataset may show a difference. An additional advantage of the XGBoost approach is its ability to provide feature importance scores, which offers insight into which modality contributes more to the final prediction. We found that fundus photos appeared to provide more informative features than OCT scans for both NAION and IIH, whereas for healthy eyes, OCT scans contributed more than fundus photographs.

We suggest there are at least two possible explanations for this pattern. First, we had a relatively large sample of healthy OCT scans sourced from a diverse range of datasets, while for IIH and NAION, there were more fundus photographs available than OCTs. Second, OCT data is inherently more complex, consisting of volumetric scans that contain hundreds of cross-sectional images. Although OCT provides rich volumetric data, two-dimensional (2D) models benefit from a much more extensive body of research, largely because 2D imaging is more widely used across domains. As a result, DL methods for 2D image analysis are more mature, while development of 3D models has progressed more slowly due to fewer applications and less available data.

While the multimodal approach leverages the strengths of combining OCT volumes and fundus photographs, it is important to note that these imaging measurements were not originally acquired with paired data specifically in mind for a DL model. To prevent data leakage, we needed to use a significantly larger test set than typical to account for all unpaired time points of paired eyes, ensuring that no overlapping data points influenced training or validation. Consequently, this led to smaller training and validation sets compared to models using either fundus photos or OCTs alone. Despite this limitation, the multimodal model outperformed our single-modality models from prior work (93.6% for photos, 90.1% for OCTs^23^^,^^25^) trained and evaluated largely on the same datasets while requiring slightly over half the training set. However, those models were evaluated on an external validation set, while our evaluation was limited to a test set composed solely of paired data. Nonetheless, our models underscore the efficacy of incorporating multiple complementary data streams to enhance diagnostic precision, even when faced with constraints on training data size.

The ability to accurately differentiate between IIH and NAION is particularly critical given their potential for overlapping clinical presentations and divergent management strategies. While OCT provides quantitative insights into ONH swelling and associated structural changes, fundus photographs offer qualitative visualizations of disc pallor, hemorrhages, and edema. The multimodal model demonstrated enhanced diagnostic accuracy for both diseases, addressing a key challenge in neuro-ophthalmology. Additionally, the model architecture allows for effective classification even when one modality is unavailable.

The clinical potential of these findings could be substantial. Fundus photos and OCT scans are inexpensive diagnostic tools that contain a wealth of diagnostic and hypothetically predictive information within their scans. Accurate and timely differentiation of IIH and NAION can facilitate more effective patient management, reducing diagnostic errors and expediting appropriate treatment. In most cases of NAION, the acute optic disc edema is unilateral, but in a number of cases the acute presentation consists of bilateral disc edema, which can create diagnostic uncertainty for distinguishing it from other causes of optic disc edema such as raised intracranial pressure, for example. Papilledema caused by IIH can be highly asymmetric adding diagnostic confusion. For IIH, early recognition of papilledema can prevent irreversible vision loss, whereas in NAION, prompt diagnosis enables vascular risk management and minimizes further visual deterioration. Moreover, timely and accurate identification of these conditions may help avoid the need for additional expensive, invasive, or otherwise unnecessary exploratory procedures, such as MRI or lumbar puncture. Conversely, correct recognition of asymmetric papilledema rather than NAION could allow clinicians to correctly proceed quickly to neuroimaging and CSF sampling, potentially diagnosing life-threatening conditions earlier.

Additionally, the inclusion of healthy controls in the analysis highlights the models’ utility in screening and triage, particularly in settings where specialized expertise is unavailable. This potential will be increased as we expand the models to include other causes of ONH edema (e.g., uveitis, optic neuritis, vitreous traction, hypotony) or ONH elevation including congenital anomalies and drusen of the optic nerve head. This methodology can also readily incorporate outputs from other data sources, including both DL and non-DL inputs from macular scan models, visual field testing, or other clinical assessments. Importantly, the approach is also flexible, as it does not require all modalities to be present. A participant with a fundus photo and an ONH OCT but no macular scan can input their findings such that the model can still produce an accurate and clinically useful prediction. We anticipate further clinical utility as we include a wider variety of disorders that elevate or swell the ONH.

This study has limitations. The inclusion of data from specific clinical trials and observational cohorts, although valuable for standardizing disease characterization, may limit generalizability to broader populations. Differences in demographic distributions between the disease groups and controls, such as the predominance of young overweight women in IIH and older men in NAION, may introduce bias. A potential confounder in this study is body mass index (BMI), as data from the University of Iowa athlete cohort and the IIH group involve participants of similar age. Because BMI was not collected, this variable could not be controlled for. Although, several studies have reported an inverse relationship between higher BMI and subfoveal choroidal thickness in otherwise healthy individuals. It's possible that in these cases, our model may be exploiting these BMI-linked choroidal features.^36^^,^^37^ However, our prior foundational work^23^ showed that among cases of intracranial hypertension secondary to other causes, who were more closely matched in age and demographics to the NAION group, differences in OCT model performance remained minimal. These findings held when controlling for race as well. Another consideration is that our paired healthy sample eyes substantially were derived from the QRK207 trial's fellow eyes that did not have NAION. Although these eyes represent variations of normal optic discs, they have a small cup-to-disc ratio and are at a higher risk of developing NAION compared to individuals without acute NAION. Although the fellow eyes were screened to exclude NAION at enrollment, its acute or subacute development during the one-year study period, although unlikely, remains plausible. It is also possible that participants with same-day imaging from both modalities systematically differed from those with unpaired measurements, as paired measurements (comprising only 29% of OCTs and 28% of photos) were predominantly allocated to the test set rather than the training or validation sets. However, the similarity in evaluation metrics between the validation set (primarily unpaired) and the test set (entirely paired) suggests this is unlikely. Last, we fit our XGBoost model within the test set via cross-validation, effectively treating the test set as a validation set rather than an independent holdout. Although this enabled us to maximize the utility of a relatively limited dataset, it may overestimate model generalizability. Partitioning off a separate untouched test set would have substantially reduced the data available of an already partitioned dataset, making robust assessment difficult. Larger datasets will be important to validate the performance of this fusion approach on an independent test set.

Future efforts should aim to validate both these models on larger, more diverse datasets of ONH swelling to improve their generalizability. Integration of additional modalities such as best corrected visual acuity, visual field testing, OCT angiography, or OCT scans of other clinically relevant areas could further improve diagnostic accuracy by providing complementary structural information. Another direction could involve developing a joint model that takes both fundus photographs and OCT scans as simultaneous inputs, allowing it to cross-reference features between modalities for potentially greater diagnostic performance. However, this approach would reduce flexibility, as it would require both imaging types to be available at inference time. Moreover, refining the fusion methods to identify the most informative features from each modality may offer new insights into the structural changes associated with ONH swelling disorders. Finally, the development of real-time, user-friendly diagnostic tools incorporating these models has the potential to streamline clinical workflows and improve patient outcomes.

This study underscores the power of multimodal imaging in enhancing diagnostic accuracy for conditions associated with optic nerve head swelling. By integrating structural and qualitative data from OCT and fundus photography, our models demonstrated superior performance in differentiating IIH, NAION, and healthy eyes. These findings represent a step forward in leveraging AI-driven multimodal imaging for neuro-ophthalmologic conditions, offering both diagnostic precision and potential insights into disease mechanisms.
